# Rewiring the Regenerated Zebrafish Retina: Reemergence of Bipolar Neurons and Cone-Bipolar Circuitry Following an Inner Retinal Lesion

**DOI:** 10.3389/fcell.2019.00095

**Published:** 2019-06-06

**Authors:** Timothy E. McGinn, Carlos A. Galicia, Dylan C. Leoni, Natalie Partington, Diana M. Mitchell, Deborah L. Stenkamp

**Affiliations:** ^1^Department of Biological Sciences, University of Idaho, Moscow, ID, United States; ^2^Department of Biology, Brigham Young University-Idaho, Rexburg, ID, United States

**Keywords:** retina, regeneration, zebrafish, circuitry, connectome, photoreceptor, retinal bipolar cell, birthdating

## Abstract

We previously reported strikingly normal morphologies and functional connectivities of regenerated retinal bipolar neurons (BPs) in zebrafish retinas sampled 60 days after a ouabain-mediated lesion of inner retinal neurons (60 DPI) (McGinn et al., 2018). Here we report early steps in the birth of BPs and formation of their dendritic trees and axonal arbors during regeneration. Adult zebrafish were subjected to ouabain-mediated lesion that destroys inner retinal neurons but spares photoreceptors and Müller glia, and were sampled at 13, 17, and 21 DPI, a timeframe over which plexiform layers reemerge. We show that this timeframe corresponds to reemergence of two populations of BPs (PKCα+ and *nyx::mYFP*+). Sequential BrdU, EdU incorporation reveals that similar fractions of PKCα+ BPs and HuC/D+ amacrine/ganglion cells are regenerated concurrently, suggesting that the sequence of neuronal production during retinal regeneration does not strictly match that observed during embryonic development. Further, accumulation of regenerated BPs appears protracted, at least through 21 DPI. The existence of isolated, *nyx::mYFP*+ BPs allowed examination of cytological detail through confocal microscopy, image tracing, morphometric analyses, identification of cone synaptic contacts, and rendering/visualization. Apically-projecting neurites (=dendrites) of regenerated BPs sampled at 13, 17, and 21 DPI are either truncated, or display smaller dendritic trees when compared to controls. In cases where BP dendrites reach the outer plexiform layer (OPL), numbers of dendritic tips are similar to those of controls at all sampling times. Further, by 13–17 DPI, BPs with dendritic tips reaching the outer nuclear layer (ONL) show patterns of photoreceptor connections that are statistically indistinguishable from controls, while those sampled at 21 DPI slightly favor contacts with double cone synaptic terminals over those of blue-sensitive cones. These findings suggest that once regenerated BP dendrites reach the OPL, normal photoreceptor connectomes are established, albeit with some plasticity. Through 17 DPI, some basally-projecting neurites (=axons) of regenerated *nyx::mYFP*+ BPs traverse long distances, branch into inappropriate layers, or appear to abruptly terminate. These findings suggest that, after a tissue-disrupting lesion, regeneration of inner retinal neurons is a dynamic process that includes ongoing genesis of new neurons and changes in BP morphology.

## Introduction

A major challenge in the treatment of human neurodegenerative diseases and trauma affecting the central nervous system is that mammals do not regenerate neurons lost to such conditions, but instead launch a gliotic response that results in tissue scarring (Pekny et al., 2014; Okada et al., 2018), and nervous tissue function is not restored. In contrast, teleost fish respond to neuronal injury in a manner that not only replaces the lost neurons, but also restores tissue function (Mensinger and Powers, 1999, 2007; Madelaine and Mourrain, 2017; Ma et al., 2017; McGinn et al., 2018). The eye's neural retina, a component of the central nervous system, provides an accessible system for studying these differences in mammals vs. fish (Wan and Goldman, 2016), for identifying factors and conditions that favor regeneration, and for potentially delivering future clinical applications in the treatment of retinal diseases and injuries that would otherwise result in loss of vision.

Vertebrate retinas are highly conserved, with light-sensing rod and cone photoreceptors organized in an outer nuclear layer (ONL), and which transmit information to bipolar cells in the inner nuclear layer (INL), which in turn transmit signals to the output neurons of the retina, the retinal ganglion cells, located in the ganglion cell layer (GCL). Horizontal cells (also in the INL) and amacrine cells (in the INL and GCL) modulate this information flow. The zebrafish animal model has been particularly enlightening toward our understanding of retinal regeneration (Stenkamp, 2015; Wan and Goldman, 2016; Ail and Perron, 2017). In zebrafish, injuries that target all neurons (Sherpa et al., 2008), only photoreceptors (Kassen et al., 2007; Nagashima et al., 2013; Ranski et al., 2018), only inner retinal neurons (INL and GCL; Fimbel et al., 2007; Sherpa et al., 2014), are regionally specific (Fausett and Goldman, 2006), or target selected specific cell types (Fraser et al., 2013; D'Orazi et al., 2016; White et al., 2017), all result in a regenerative response that replaces these missing neurons. Studies making use of these injuries have revealed that the major glial cell type of the retina, the Müller glia, is the stem cell source of regenerated retinal neurons in the zebrafish (Fausett and Goldman, 2006; Bernardos et al., 2007; Nagashima et al., 2013). Several regulatory factors have been identified that promote or are necessary for Müller glia to re-enter the cell cycle and generate neural progenitors (Fausett et al., 2008; Qin et al., 2011; Ramachandran et al., 2011a,b; Lenkowski et al., 2013; Nagashima et al., 2013; Nelson et al., 2013), which then proliferate and produce the regenerated retinal neurons (Nagashima et al., 2013; Gorsuch and Hyde, 2014; Powell et al., 2016). Some of this knowledge has already been applied in strategies for coaxing the Müller glia of mouse retina to generate retinal neurons (Ahmad et al., 2011; Hyde and Reh, 2014; Jorstad et al., 2017; Yao et al., 2018).

A widely anticipated obstacle for the future application of endogenous regeneration strategies, as well as for the use of transplantation approaches to treat retinal disorders, is ensuring that the replacement neurons establish synaptic communication with the appropriate pre- and/or post-synaptic partners (Angueyra and Kindt, 2018). Indeed, teleost fish not only regenerate lost neurons, these regenerated neurons restore visual functions indicating successful rewiring (Mensinger and Powers, 1999, 2007; Sherpa et al., 2008, 2014; McGinn et al., 2018). Further, this restoration of visual function is possible even when pre- and post-synaptic partners are simultaneously destroyed (Mensinger and Powers, 1999, 2007; Sherpa et al., 2008, 2014; McGinn et al., 2018). Recently, Yao et al. (2018) showed that such successful rewiring is possible in a mouse model for rod photoreceptor degeneration, in that new rods regenerated by genetically altered Müller glia can transmit signals that are propagated through the visual system. However, this promising and exciting finding does not negate the obstacle of rewiring in mammalian retinal disorders, because in these disorders the retinal environment is altered by an inflammatory response (Arroba et al., 2018), neuronal remodeling takes place (Genove et al., 2014), gliotic scarring may block access to synaptic partners (Genove et al., 2014), and multiple neuronal cell types may be destroyed, damaged, or their function altered (Narayan et al., 2016).

The zebrafish retina again appears to represent a system that demonstrates the ability to overcome these obstacles. The damaged zebrafish retina also displays features that could be considered obstacles similar to those in damaged mammalian retina. The damaged zebrafish retina experiences an infiltration of microglia and macrophages (Mitchell et al., 2018), retains the potential for a gliotic response (Morris et al., 2008; Thomas et al., 2016), undergoes retinal remodeling (Saade et al., 2013), and the regenerating/regenerated zebrafish retina shows abnormal patterns of lamination of the retinal layers (Sherpa et al., 2014), and of the two-dimensional organization of neuronal classes within each layer (Vihtelic and Hyde, 2000; Stenkamp and Cameron, 2002). Regenerating zebrafish retinas also produce supernumerary neurons, and produce other neuronal types in addition to those lost to the initial injury (Sherpa et al., 2008, 2014; Powell et al., 2016). Despite these apparent obstacles, however, regenerated zebrafish retina is functional, as measured by behavioral assays for vision (Sherpa et al., 2008, 2014), and a physiological assay, the electroretinogram (ERG) (McGinn et al., 2018), suggesting at least some degree of accurate rewiring. Consistent with this idea, a recent report by D'Orazi et al. (2016) noted minimal errors in connectivity patterns of regenerated retinal bipolar (BP) neurons after cell-selective lesion in larval zebrafish. Further, we recently reported that regenerated BPs of adult zebrafish also establish essentially normal cone connections, dendritic and axonal arbor morphologies, and axon terminal stratification patterns following a tissue-disrupting lesion that destroys inner retinal neurons (McGinn et al., 2018). These studies strongly suggest that the process of rewiring regenerated retinal neurons is successful in the zebrafish, regardless of the environmental conditions created by damage.

Do newly-regenerated BPs immediately display normal morphologies and connectivities, or are these features differentiated later? When do regenerated BPs emerge relative to their synaptic partners, when partners are also destroyed and regenerated? Does this match the sequence of synaptic partner generation seen during embryonic development? When do regenerated BPs show mature morphologies and accurate connectivities? This information will be of immense utility for therapeutic applications in which transplanted or regenerated retinal neurons must accomplish the same in an injured human retina (Angueyra and Kindt, 2018). As the next step toward answering these questions and achieving this goal, in the present study we describe the early stages of the BP regenerative process following a tissue-disrupting injury that selectively destroys inner retinal neurons. We find that regenerated BPs appear from 13 to 21 days post-injury (DPI), coincident with the reemerging plexiform layers (Fimbel et al., 2007), and birthdating indicates that, in contrast to the developmental sequence, these BPs are regenerated concurrently with their postsynaptic partners (amacrine and ganglion cells). The dendritic trees and axonal arbors of some newly regenerated BPs remain immature or highly abnormal at 13 and 17 DPI, but all sampled regenerated BPs show normal dendritic tree morphologies by 21 DPI. The generation and morphological differentiation of regenerated BPs appears distinctive in many ways in comparison with these processes during embryonic development, underscoring the need to further investigate this process.

## Materials and Methods

### Animals and Retinal Lesioning

#### Animals

Zebrafish (both sexes) used in this study were either of a wild-type strain originally obtained from Scientific Hatcheries (SciH, now Aquatica Tropicals), or were transgenic for one or both of the following transgenes: *nyx::mYFP, sws2:mCherry*. The *nyx::mYFP* transgenics are a line in which the *nyctalopin* (*nyx*) promoter drives Gal4, likely co-integrated with a construct in which the UAS enhancer drives expression of membrane-associated yellow fluorescence protein (mYFP) (Schroeter et al., 2006). In the adult *nyx::mYFP* zebrafish, a subpopulation of ON and Mixed (ON/OFF) retinal bipolar neurons (BPs) expresses YFP (McGinn et al., 2018). The *sws2:mCherry* transgene results in mCherry expression in blue-sensitive (SWS1) cone photoreceptors (Sifuentes et al., 2016). Zebrafish were maintained according to Westerfield (Westerfield, 2007) in monitored, recirculating system water, on a 14:10 h light:dark cycle. All procedures using animals were approved by the University of Idaho Institutional Animal Care and Use Committee.

#### Retinal Lesioning

Retinas of adult fish were chemically lesioned as previously described (Fimbel et al., 2007; Nagashima et al., 2013; Sherpa et al., 2014; McGinn et al., 2018; Mitchell et al., 2018, 2019). Briefly, corneas of anesthetized fish were perforated with a sapphire blade to introduce a Hamilton syringe containing 40–70 μM ouabain (inhibitor of the Na^+^/K^+^ ATPase) in sterile saline. The injected volume (~0.5 μL) resulted in an estimated intraocular concentration of 2 μM. Uninjected contralateral eyes were used as controls. This lesioning strategy has been demonstrated to result in death of inner retinal neurons, but spares photoreceptors, Müller glia, and microglia (Fimbel et al., 2007; Sherpa et al., 2014; Mitchell et al., 2018). Müller glia respond to this damage by re-entering the cell cycle and generating progenitors that in turn proliferate and generate the neuronal cell types lost to the lesion (Fimbel et al., 2007; Nagashima et al., 2013), ultimately restoring function (Sherpa et al., 2014; McGinn et al., 2018). The loss of BPs, survival of cones, and regeneration of BPs was monitored by observation of retinas of live, anesthetized fish using a Nikon SMZ 1500 epifluorescence stereomicroscope.

### Comparative Cell Birthdating During Retinal Regeneration

Sequential systemic exposure to bromodeoxyuridine (BrdU) and ethynyldeoxyuridine (EdU) was carried out according to Nagashima et al. (2013). For these experiments, wild-type zebrafish (*n* = 3 per condition) were transferred to 250 mL beakers containing 1.0 mM BrdU, and after a defined exposure period, were transferred to 125 μM EdU. The exposure protocols used in this study were: 4–6 DPI BrdU followed by 6–8 DPI EdU, and 6–9 DPI BrdU followed by 9–13 DPI EdU. Solutions were refreshed once daily during these exposures, and fish were fed after each refreshing.

### Tissue Processing and Immunofluorescence

Procedures were similar to those of McGinn et al. (2018). Briefly, dark-adapted fish were humanely sacrificed with MS-222, and eyes enucleated with forceps. For retinal cryosections, eyes were fixed with 4% paraformaldehyde containing 5% sucrose in phosphate buffer (pH = 7.4) for 1 h. Eyes were cryoprotected, embedded, and frozen in a 1:2 combination of Tissue-Tek OCT (Sakura Finetek):phosphate-buffered 20% sucrose, and then cryosectioned at 5 μm on a Leica CM3050 cryostat. For whole retinal flat mounts, corneas were perforated with dissecting scissors to gain access for removal of the lens. The sclera and RPE were removed with forceps and the freed retinas were rinsed in cold phosphate-buffered (pH = 7.4) saline (PBS) or HEPES. Four radial incisions helped to flatten each retina, and they were then fixed in phosphate-buffered 4% paraformaldehyde containing 5% sucrose for 1 h. Retinas were then washed three times in PBS for 30 min each.

For immunofluorescence detection of antigens, 5 μm sectioned retinas were rinsed with PBS with 0.5% triton (PBST) and then blocked for 1 h with 20% normal goat serum and 0.1% sodium azide, diluted in PBST, at room temperature. Primary antibodies were diluted in antibody dilution buffer (PBST, 1% normal goat serum, and 0.1% sodium azide), and applied to sections, which were then incubated overnight at 4°C. Sections were washed with PBS three times, for 20 min each, incubated with secondary antibodies diluted in antibody dilution buffer (PBST with 1% normal goat serum and 0.1% sodium azide) and 4.25 μM DAPI overnight at 4°C, washed with PBST for 30 min and mounted in Vectashield (Vector Laboratories) or Fluoromount-G (SouthernBiotech). For detection of BrdU and EdU, an antigen retrieval step was incorporated (1:1 solution of 4N HCl:PBST) prior to the blocking step. Whole retinas were stained with primary antibodies in antibody dilution buffer for 1–2 weeks at 4°C with constant gentle agitation, washed with PBS three times (20 min each), and then incubated with secondary antibodies diluted in antibody dilution buffer and 4.28 μM DAPI at 4°C for another week. After staining, retinas were washed with PBS three times for 20 min each.

Primary antibodies that were used in this study, and their sources and dilution are as follows. ZPR1 is a mouse monoclonal that labels cone arrestin3a, staining both the red- and green-wavelength sensitive members of the double cone pair (Renninger et al., 2011) (ZIRC; 1:200). Anti-protein kinase Cα (PKCα) is a rabbit polyclonal antibody originally produced to target the C-terminus of human PKCα, and labels a subpopulation of BP neurons (Suzuki and Kaneko, 1990) (Santa Cruz Biotechnology; SC-10800 1:200). Anti-HuC/D is a rabbit polyclonal antibody that stains inner retinal neurons, primarily ganglion cells and amacrine cells (abcam, Eugene, OR; 1:100). Anti-BrdU is a mouse monoclonal antibody (Invitrogen, Carlsbad, CA; 1:200). EdU was detected using the “Click-It” reaction kit (Invitrogen). Secondary antibodies (Jackson ImmunoResearch, West Grove, PA, all 1:200) used in this study were donkey anti-mouse AlexaFluor 647, donkey anti-rabbit Cy3, donkey anti-rabbit TRITC, and donkey anti-rabbit FITC.

### Imaging and Quantification of Neurons in Cryosections

**Figure 1:**
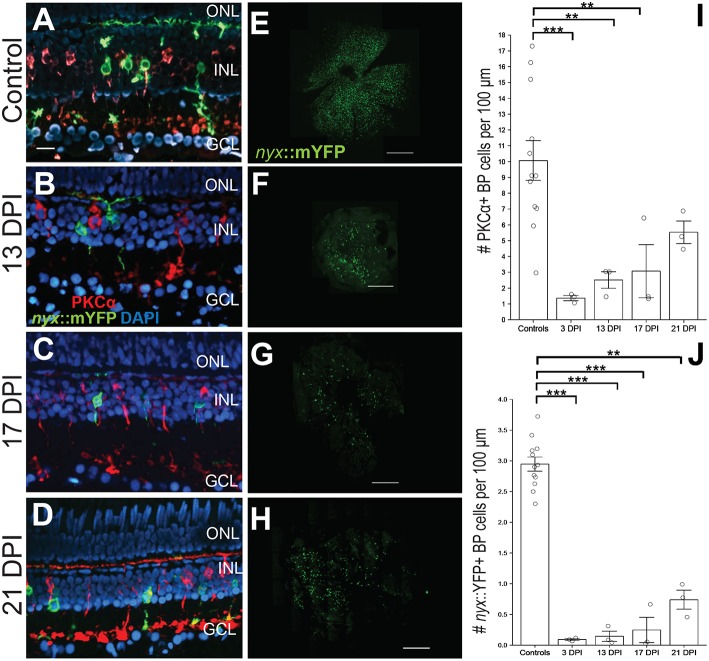
Emergence of identifiable retinal bipolar (BP) neurons following a chemical lesion selective to the inner retina. **(A)** PKCα+ and *nyx::mYFP*+ BP cell bodies occupy the inner nuclear layer (INL), with dendritic trees in the OPL and axon terminals in the inner plexiform layer of control retinas. **(B–D)** New PKCα+ and *nyx::mYFP*+ BPs reappear over the time frame of 13 days post-injury (DPI) **(B)**, 17 DPI **(C)**, and 21 DPI **(D)**, and have recognizable apical processes (dendrites) and basal processes (axons). **(E–H)** Retinal flat mounts of control **(E)**, and regenerated, *nyx::mYFP* retinas at 13 DPI **(F)**, 17 DPI **(G)**, and 21 DPI **(H)** showing distributions of regenerated *nyx::mYFP*+ BP neurons (*n* = 11) controls, six 13 DPIs, five 17 DPIs, and five 21 DPIs prepared as whole mounts (Supplementary Table 1) and visually inspected for overall distribution of *nyx::mYFP* BPs. Three controls, two 13 DPIs, three 17 DPIs, and three 21 DPIs were imaged as in **(E-H)**. High resolution images of these retinas are provided in Supplementary Figure 1. **(I)** Numbers of PKCα+ BPs at 3 (McGinn et al., 2018), 13, and 17 DPI were significantly different from controls (**p < 0.01; ***p < 0.001), while there was no statistically significant difference between controls and 21 DPI (*p* = 0.246), or for any other *post-hoc* pairwise analysis (Kruskall-Wallis, Conover's *post-hoc* analysis; graph shows means ± SEM). **(J)** Numbers of *nyx::mYFP*+ BPs remained significantly reduced at 3, 13, 17, and 21 DPI (**p < 0.01; ***p < 0.001), but there was no statistically significant difference for any other *post-hoc* pairwise analysis (Kruskall-Wallis, Conover's *post-hoc* analysis; graph shows means ± SEM). Scale bar in **A** (applies to **A–D**) = 20 μm. Scale bars in **E-H** each = 200 μm.

Sections were imaged using an Andor Zyla 5.5 sCMOS camera connected to a Nikon Ti inverted microscope with a Yokogawa spinning disk using a 20x 0.75NA air objective, a 40x 1.3NA oil immersion, a 60x 1.40NA oil, or a 100x 1.45NA oil immersion objective. BP neurons were counted in PKCα-stained retinal cryosections of *nyx::mYFP* fish (n=3 per condition, with 9 contralateral controls), by identifying PKCα+, DAPI+ cell bodies, *nyx::mYFP*+, and DAPI+ cell bodies, within 5 μm-thick radial sections imaged at 20x. To quantify BPs in cryosections, only cell bodies showing both DAPI and a BP-specific marker were counted. To quantify inner retinal neurons following cell birthdating by BRDU/EdU immersion (described above), BP neurons (PKCα+) and HuC/D+ neurons were counted in retinal cryosections following staining for PKCα, BrdU, and EdU, or for HuC/D, BrdU, and EdU. BPs and HuC/D+ neurons that incorporated BrdU or EdU were counted by identifying cell-specific marker+, nucleotide+ cell bodies. For both analyses, sections were sampled over the regions between the larval remnant (Allison et al., 2010) and the extreme periphery. We counted every 5th section to avoid double-counting. We note that, if the nuclei of newly regenerated BPs were smaller than those of control BPs, this counting method may under-sample neurons in the regenerated retinas (Coggeshall and Lekan, 1996). However, to our knowledge (and see Figure 1), nuclei of regenerated retinal neurons do not appear substantially different in size in comparison with those of their control counterparts.

### Imaging and Morphometric Analyses of Bipolar Neurons in Whole Retinas

Whole retinas were mounted in Fluoromount-G or Vectashield Hardset, with the GCL facing the coverslip, and using electrical tape as a spacer between the slide and coverslip. Retinas were imaged with the Andor/Nikon system described above using a 60x 1.2NA water immersion objective, with Immersol W 2010 (Zeiss). Images were collected from multiple locations throughout the retina when possible, but for some retinas only a very few *nyx::mYFP*+ neurons were found. Multiple image stacks were collected using 0.3 μm z-steps through the entire thickness of the retina. As in McGinn et al. (2018), each stack was 114.62 μm wide by 164.88 μm high, or 1028x1522 pixels, resulting in a scale of 9.23 pixels/μm.

Image stacks were analyzed in Fiji software (ver 1.51d) (Fiji, RRID:SCR_002285) (Schindelin et al., 2012). Selected, *nyx::mYFP*+ cells were traced with the Simple Neurite Tracer (SNT) plugin (Longair et al., 2011). Each BP was visually assessed for meeting the following set of criteria defining a “stereotypical appearance” of a BP: Cell body within the INL, having a single apical process with multiple branches within the outer plexiform layer (OPL), and having a single basal process with terminals in the IPL, and in general resembling one of the type specimens identified by Li et al. (2012). Only cells showing a clear soma, dendrite, and axon (or other projections) within the field of view were traced (numbers of traced neurons are provided in Supplementary Table 1).

Traced neurons were filled using the fill option in SNT. Because SNT was not designed to show larger 3D objects such as a cell body, only portions of cell bodies were traced and filled, while the axons and terminals, and dendritic trees were traced and filled in their entirety. The axon, soma, and dendrite of each BP were each converted in an image stack and imported into ImageJ's 3D viewer as surfaces. Each neuron was saved as a separate file in the OBJ format. OBJ files were imported into 3ds Max 2016, placed together by their respective time point and then rendered. Numbers of neurons subjected to surface rendering are provided in Supplementary Table 1.

Sholl analysis was performed on traced BPs to determine the characteristics of individual dendritic trees and axonal branching patterns. For BP dendrites, Sholl analysis was done for traced neurons that displayed a clear, apically-projecting primary dendrite, showed branching, and had dendritic spreads sufficient for the analysis to return meaningful values (e.g., dendrites with only one very small branch would be excluded). For BP axons, Sholl analysis was done for traced neurons that displayed a clear, basally-projecting axon that reached the IPL, showed branching within the IPL, and could be traced for its entirety. The Sholl Fiji plugin utilized several different variants of the original Sholl Analysis (Sholl, 1953) in order to test for differences in neuronal architecture as the retina regenerates. A series of concentric shells (spheres in this case) were created around a centerpoint. For dendrites used in this study the point at which the primary dendrite branches was considered the center. For axons the point where the primary axon had the largest number of branches was considered the centerpoint. The software then counted how many branches crossed or intersected at each particular enclosing shell. Numbers of neurons subjected to Sholl analysis of dendritic trees and axonal arbors are provided in Supplementary Table 1.

Cone-*nyx::mYFP*+ BP contacts were identified using the strategy described by McGinn et al. (2018). In brief, raw.ND2 images were imported into Fiji, and YFP+ dendrites were traced with SNT, and filled in using the “fill” option. Independent stacks were generated for each dendrite, and re-merged with ZPR1+ fluorescence (double cones) and *sws2:mCherry* fluorescence (blue-sensitive cones) as separate channels. We next generated a partial Z-projection containing only cone synaptic pedicles and the untraced dendritic tree of the *nyx::mYFP*+ BP under analysis. BPs with highly truncated dendritic trees were excluded from this analysis. ZPR1+ vs. mCherry+ cone synaptic pedicles were outlined as separate colors using the region of interest tool. The total number of dendritic tips for each analyzed BP was counted, and each dendritic tip showing contact with an outlined cone pedicle was recorded. Numbers of primary apical (dendritic) and basal (axonal) processes were counted for each traced, *nyx::mYFP*+ BP.

Each BP was visually assessed for meeting the following set of criteria defining a “stereotypical appearance” of a BP: Cell body within the INL, having a single apical process with multiple branches within the OPL, and having a single basal process with terminals in the IPL, and in general resembling one of the type specimens identified by Li et al. (2012).

Some selected neurons were traced using Filament Tracer, in Imaris version 8.2.0 (Bitplane), an alternative tracing strategy that allows visualization of traced neurons with a background grid and from multiple views. Neurons selected for this analysis were those that were physically isolated from other *nyx::mYFP*+ neurons and had morphologies that could not be as easily appreciated using the viewpoints available following use of SNT. Multiple views were collected in 3D View in order to demonstrate how the neurons were positioned within the retinal region represented by the confocal stack. Images showing the top and side views were collected in the Slice View in order to show partially projected top down and side views of neurons. In order to visualize the traced neurons in this module, a new color channel was created by selecting the “create channel from filament” option from the traced axon, and pseudocolored red. Another color channel was created for the traced dendritic tree and pseudocolored green.

### Statistics

Numbers of PKCα+ and *nyx::mYFP*+ cells/100 μm of retinal section were compared across samples, including the data from 3 DPI samples reported in McGinn et al. (2018), using a Kruskal-Wallis test, followed by a Conover's test for multiple comparisons of independent samples. For statistical analysis of morphometric data of individual BP neurons, data were imported into R Studio (ver 0.99.903) (R Project for Statistical Computing) using R (ver 3.3.1) for statistical analysis. ANOVAs and Kruskal-Wallis tests were used for parametric and most non-parametric data, respectively. Since none of the parametric data showed statistical significance (*p* < 0.05) as measured by a one-way ANOVA, no *post-hoc* tests were conducted. For any Kruskal-Wallis test outcome that had a *p*-value < 0.05, a *post-hoc* test was done using a Wilcoxon–Mann–Whitney test with a false discovery rate *p*-value adjustment. A generalized linear model with a Poisson distribution was applied for analysis of connectivity patterns, and any comparisons having *p*-values < 0.05 were considered significant. Sample sizes were *n* = 3 for quantifications in cryosections (with 12 contralateral controls for PKCα+ and *nyx::mYFP*+ BP counting); Supplementary Table 1 contains the numbers of neurons subjected to morphometric analyses.

## Results

### Emergence of Regenerated BPs Following Ouabain Lesion

We previously documented strikingly normal morphologies of regenerated BPs in retinas sampled at 60 days after a ouabain-mediated lesion of inner retinal neurons (McGinn et al., 2018). This lesioning strategy has been verified to destroy neurons of the inner retina by 3 days post-injury (DPI), sparing Müller glia, photoreceptors, and very few horizontal cells (Fimbel et al., 2007; Nagashima et al., 2013; Sherpa et al., 2014; McGinn et al., 2018; Mitchell et al., 2018). At 60-80 DPI after this lesion, functional recovery of vision is evident by behavioral and physiological analyses (Sherpa et al., 2014; McGinn et al., 2018). To gain insights into the process of the formation of BP dendritic trees and axonal branching patterns during regeneration, zebrafish subjected to such a lesion were sampled at 13, 17, and 21 DPI. This timeframe was selected in part because neurons of the layers that were destroyed (INL and GCL) are regenerated by this time, and plexiform layers re-emerge (Fimbel et al., 2007; Mitchell et al., 2018). Furthermore, microglia remain active over this timeframe (Mitchell et al., 2018), and their involvement in synaptic remodeling has been documented in other contexts (Schafer et al., 2012). We therefore reasoned that 13–17 DPI may represent a period of BP reemergence as well as synaptogenesis and refinement of neuronal processes during retinal regeneration. The loss of BPs and their regeneration was confirmed in lesioned, *nyx::mYFP*; *sws2:mCherry* transgenic zebrafish by observation of eyes of live, anesthetized fish (*n* = 10) at 3, 5, 10, 11, 12, and 13 DPI, by epifluorescence stereomicroscopy. These observations confirmed loss of mYFP+ signal (corresponding to BPs), and the continued presence of distinctive rows of mCherry+ cells (blue-sensitive, *sws2*-expressing cones) within retinas at 3–5 DPI (data not shown). Uninjected, contralateral eyes retained expression of both transgenic reporters. In lesioned fish, new YFP+ fluorescence was again visible for the first time at 13 DPI, and so we focused our detailed analyses beginning at this timepoint.

We first documented the recovery of *nyx::mYFP*+ BPs [a population including ON BPs and mixed ON/OFF BPs (Schroeter et al., 2006; McGinn et al., 2018)], and PKCα+ BPs [ON BPs (Suzuki and Kaneko, 1990; Schroeter et al., 2006; McGinn et al., 2018)] in regenerated retinas over 13–21 DPI (Figure 1). We previously demonstrated that these are separate populations of BPs in adult zebrafish retinas, with minimal overlap (McGinn et al., 2018) (Figure 1A). Consistent with our observations in live, anesthetized fish, small numbers of PKCα+ and of *nyx::mYFP*+ BPS were observed in retinal cryosections of fish sampled at 13 DPI (Figure 1B) and 17 DPI (Figure 1C), and these BP cell types appeared more abundant in cryosections of fish sampled at 21 DPI (Figure 1D). The restoration of these cell types was quantified in the cryosections and numbers were compared among groups, including previously-reported PCKα+ BP numbers at 3 DPI (McGinn et al., 2018), when considerable damage has occurred, and with contralateral controls. PKCα+ BPs were present, but were reduced in numbers at 13 and 17 DPI in comparison with controls, while there was no statistical significance regarding a difference between PKCα+ BP numbers at 21 DPI in comparison with controls (Figure 1I; Kruskal-Wallis with Conover's *post-hoc*), suggesting restoration of these BP neurons at this timepoint. However, *post-hoc* analyses of PKCα+ BP numbers for all other pairwise comparisons, including in comparison to numbers at 3 DPI, did not indicate statistically significant differences (Figure 1I). We note that the small sample number at each timepoint likely limits conclusions from this analysis. The *nyx::mYFP*+ BPs also appear over this time frame, in a similar temporal pattern, but at lower densities compared to PKCα+ BPs. The *nyx::mYFP*+ BPs remained reduced in numbers at 13, 17, and 21 DPI in comparison with controls (Figure 1J; Kruskal-Wallis with Conover's *post-hoc*). Similar to the findings for PKCα+ BPs, *post-hoc* analyses of all other pairwise comparisons, including comparison to numbers at 3 DPI, did not indicate statistically significant differences of *nyx::mYFP*+ BPs (Figure 1J). Again, small sample sizes at all timepoints likely limit conclusions from this analysis. Findings for *nyx::mYFP* BPs should be interpreted with even further caution, because the low numbers of newly regenerated *nyx::mYFP*+ BPs may be in part related to transgene silencing (Goll et al., 2009) or delay in expression of the fluorescent reporter. The *nyx::mYFP*+ retinas were also sampled as whole mounts at 13, 17, and 21 DPI by confocal microscopy (Figures 1E–H; Supplementary Figure 1). BPs appeared in central and peripheral retina, and were visible in all quadrants of flat mounted retinas, suggesting no biases favoring a particular location of regenerated *nyx::mYFP* BPs (Figures 1E–H).

### Relative Birthdating of Regenerated BPs and Other Retinal Neurons

**Figure 2:**
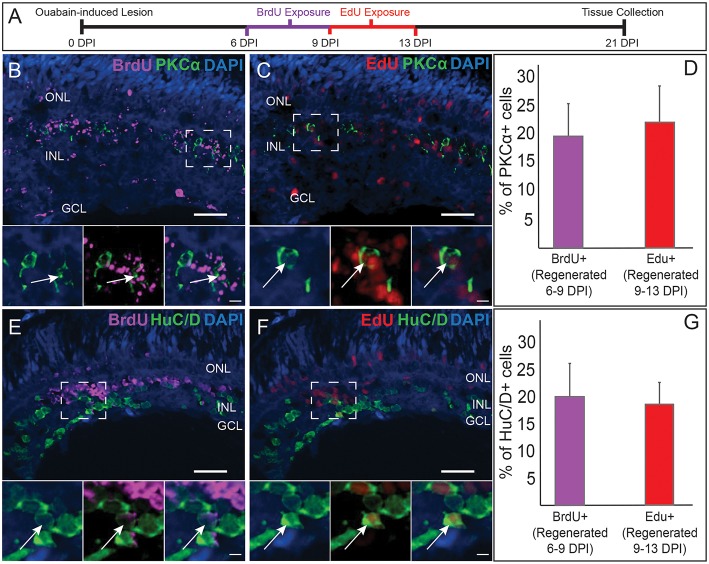
Comparative birthdating of regenerated PKCα+ bipolar (BP) neurons and HuC/D+ amacrine and ganglion cells following a chemical lesion selective to the inner retina. **(A)** Timeline and experimental design; DPI, days post-injury. **(B)** Some regenerated PKCα+ (green) BPs incorporated BrdU (magenta) (6–9 DPI). Higher magnification insets of boxed region show PKCα and DAPI, PKCα, and BrdU, and all channels merged. Arrows indicate BrdU+ PKCα+ BP cell. **(C)** Some regenerated PKCα+ BPs incorporated EdU (red) (9–13 DPI). Higher magnification insets corresponding to boxed region show PKCα and DAPI, PKCα and EdU, and all channels merged. Arrow indicates EdU+ PKCα+ BP cell. **(D)** Percentage of PKCα+ cells that incorporate BrdU and EdU at the indicated timeframes (means ± s.d.; *n* = 3). **(E)** Some regenerated HuC/D+ (green) neurons incorporated BrdU (magenta) (6–9 DPI). Higher magnification insets corresponding to boxed region show HuC/D and DAPI, HuC/D and BrdU, and all channels merged. Arrow indicates BrdU+ HuC/D+ cell. **(F)** Some regenerated HuC/D+ BPs incorporated EdU (red) (9–13 DPI). Higher magnification insets corresponding to boxed region show HuC/D and DAPI, HuC/D and EdU, and all channels merged. Arrow indicates EdU+ HuC/D+ cell. **(G)** Percentage of HuC/D+ neurons that incorporate BrdU and EdU at the indicated timeframes (means ± s.d.) are similar to the percentages of PKCα+ BPs that incorporated these labels. Scale bar in **B** (applies to **B,C,E,F**) = 50 μm. Scale bar in insets (applies to all insets) = 10 μm.

The quantification of regenerated BPs in cryosections, together with our previous studies showing that some regenerated BPs were labeled by BrdU exposures at either 4–7 or 6–12 DPI (McGinn et al., 2018), suggested that the regeneration of BPs is a protracted and likely asynchronous process, distinct from the rapid wave of BP generation that takes place during zebrafish embryonic development (Hu and Easter, 1999). Alternatively, or in addition, regenerating BPs may exhibit delayed and/or asynchronous expression of the markers used to identify them. To distinguish among these possibilities, we used two different, sequential BrdU-EdU exposures, and quantified PKCα+ BPs expressing either or both nuclear markers upon tissue collection at 21 DPI. Early exposures (4–6 DPI BrdU, 6–8 DPI EdU) revealed very little incorporation and retention of the BrdU label (data not shown), suggesting onset of BP regeneration begins after 6 DPI. Later exposures (6–9 DPI BrdU, 9–13 DPI EdU; Figure 2A) revealed sufficient incorporation and retention of label for quantification (Figures 2B,C). At 21 DPI, an average of ~20% of the PKCα+ BPs were BrdU+ (born 6–9 DPI), and another ~20% were EdU+ (born 9–13 DPI) (Figure 2D), consistent with steady, protracted production of this cell population. The highly limited numbers of *nyx::mYFP*+ BPs (Figure 1J) in regenerated retinas at the sampled timepoints, along with challenges related to the experimental procedure (YFP fluorescence not reliably surviving the antigen retrieval process for BrdU staining), and interpretation (outcomes related to possible transgene silencing), precluded any parallel birthdating analysis of this BP population.

We next compared the PKCα BP birthdating results with those for regenerated retinal neurons that are HuC/D+. These neurons correspond to retinal ganglion cells and amacrine cells (Kay et al., 2001), the postsynaptic partners of BPs, which are also destroyed and subsequently regenerated following ouabain injection (Fimbel et al., 2007; Sherpa et al., 2014). In zebrafish embryonic retinal development, cells of the GCL are generated prior to those of the INL (Hu and Easter, 1999), yet whether generation of these inner retinal neurons (and specifically BPs vs. their post-synaptic partners) follows similar relative timing is not yet known for regeneration. At 21 DPI, HuC/D+ cells were detected that incorporated BrdU (Figure 2E) or EdU (Figure 2F), consistent with accumulation of HuC/D+ neurons from 7 to 21 DPI (Fimbel et al., 2007). Interestingly, an average of ~20% of the HuC/D+ cells were BrdU+, and another ~20% were EdU+ (Figure 2G), very similar to the findings for the PKCα+ BPs. The HuC/D+ neuronal population that arises between 6 and 13 DPI is therefore also regenerated steadily over this timeframe, and not likely in a temporal pattern that is earlier or later than the PKCα+ BP neuronal population. The sequence of cell birth during retinal regeneration through at least 13 DPI therefore does not appear to match that observed during embryonic retinal neurogenesis, and also further supports that regeneration of inner retinal neurons following this damage paradigm is a protracted process resulting in accumulation of regenerated neurons over time (Fimbel et al., 2007; Sherpa et al., 2014). Further, and of additional importance to the present study, regenerated postsynaptic partners of the BPs are born over the same timeframe and at a similar rate as the BPs themselves.

### Morphologies of Newly Regenerated *nyx::mYFP* BPs: Qualitative Findings

**Figure 3:**
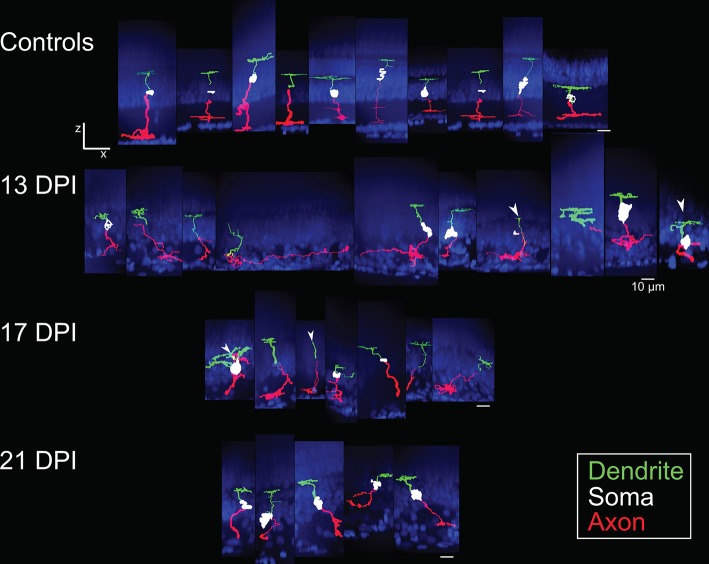
Galleries of traced *nyx::mYFP*+ retinal bipolar (BP) neurons. Neurons were traced in Simple Neurite Tracer (SNT), dendrites (green), cell bodies (white), axons (red) were pseudocolored, and then merged with images of the DAPI-stained (blue) nuclei to visualize retinal layers; merged images were then resliced to show the orthogonal views, with preservation of alignment of the traced cells and DAPI stained nuclei. For some BPs the cell body was not traced or only minimally traced due to limitations of SNT. Top row: BPs sampled from control retinas included some that were also shown in McGinn et al. (2018). Second row: BPs sampled at 13 days post-injury (13 DPI) vary in appearance, with some displaying long, wandering axons, and others displaying simple apical processes but no dendritic trees, and others presenting an apparently normal morphology. Third row: BPs sampled at 17 DPI also vary in appearance, with unusual dendritic tree structures or only simple apical processes, and others appearing normal. Bottom row: BPs sampled at 21 DPI show morphologies that more closely resembled those of control retinas. Scale bar = 10 μm.

**Figure 4:**
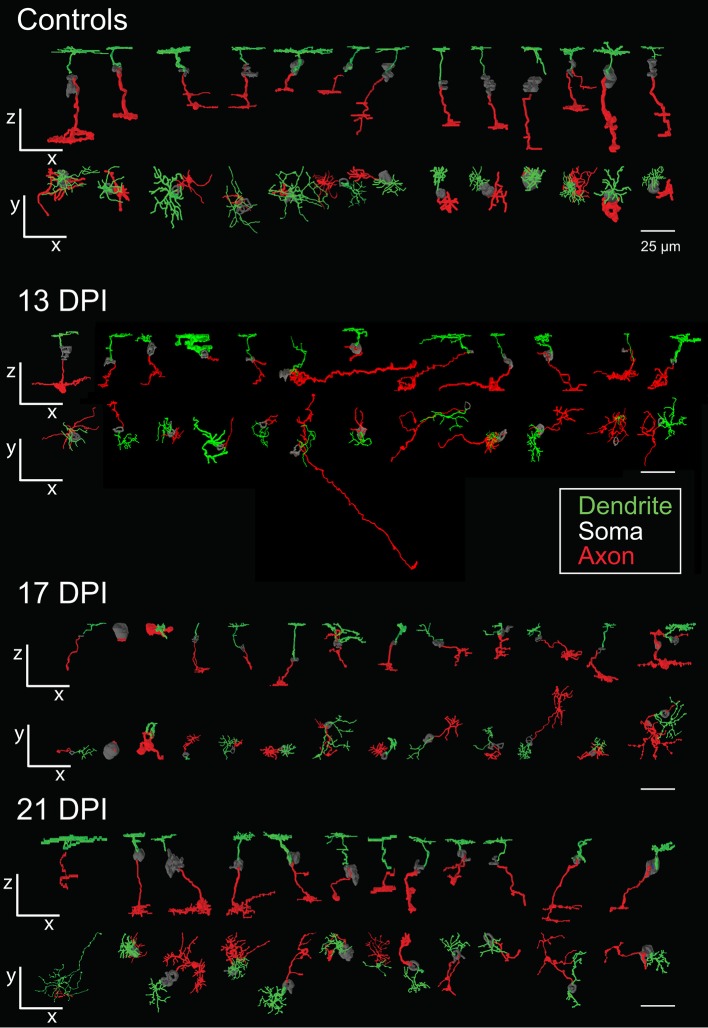
Galleries of surface rendered *nyx::mYFP*+ retinal bipolar (BP) neurons. Neurons were traced in Simple Neurite Tracer (SNT), colorized, filled, created into three-dimensional surface models, imported into 3ds Max 2016, and then entered into a single image to display overall similarities and differences of BPs. For some BPs the cell body was not traced or only minimally traced due to limitations of SNT. Each timepoint shows the rendered neurons in the x-z radial “side” views (top row) and the x-y tangential “top-down” views (bottom row) relative to the imaging plane. BPs shown from control retinas included some that were also shown in McGinn et al. (2018). Dendritic tree alignment of the seventh 13 DPI neurons is offset vertically in order to fit all into the display without overlap. Scale bars = 25 μm. DPI, days post-injury.

The sparse cellular densities of the regenerated *nyx::mYFP*+ BP neurons made them amenable to detailed morphometric analyses, because many were physically isolated from any surrounding *nyx::mYFP*+ BPs, allowing complete tracing of individual cell dendritic trees, somas, and axonal processes after imaging. Such analyses were not possible for the PKCα+ BPs, most notably within undamaged retinas, where PKCα+ BPs are very densely distributed (Figure 1I), and because the PKCα antibody did not adequately penetrate whole mounted retina tissue. Several differentiating *nyx::mYFP*+ BPs identified in whole retinas sampled at 13, 17, and 21 DPI were therefore imaged and traced (Supplementary Table 1), visualized as resliced Z-projections (Figure 3), and also visualized following surface rendering (Figure 4). For purposes of comparison, a series of control, *nyx::mYFP*+ BPs sampled from undamaged, control retinas, is also shown in each Figure (Figures 3, 4); some of these control neurons were also shown in McGinn et al. (2018). Supplementary Videos 1–5 provide rotating perspective animations of the galleries shown in Figure 4 (Videos 1-4), and of a gallery of regenerated *nyx::mYFP*+ BPs sampled at 60 DPI [Video 5; (McGinn et al., 2018)]. Note that the cell bodies of some of these BPs remained incompletely traced/filled due to limitations of the SNT plugin. The majority of sampled, regenerated *nyx::mYFP* BPs displayed apically-projecting neurites, as well as basally-projecting neurites (Figures 3, 4). For simplicity, apically-projecting neurites with terminals within or approaching the OPL are referred to as dendrites (green processes in Figures 3, 4), and basally-projecting neurites with terminals within or approaching the inner plexiform layer (IPL) are referred to as axons (red processes in Figures 3, 4). At 13 and 17 DPI, some dendritic trees did not projected fully into the OPL (one of 15 at 13 DPI, three of 13 at 17 DPI), while all of the BPs sampled at 21 DPI had dendritic trees that projected into the OPL (Figure 3; Supplementary Table 2). Some axons of BPs in 13 DPI retinas were apparently longer and meandered more than those of 17 DPI, 21 DPI, or undamaged retina, and axons of 21 DPI BPs appeared to have more complex morphologies than those sampled at earlier timepoints or from control retinas (Figures 3, 4; Supplementary Table 2). The IPL could not be readily identified in some cases, particularly at 13 DPI, as there were many nuclei present within the presumed corresponding region (Figure 3).

**Figure 5:**
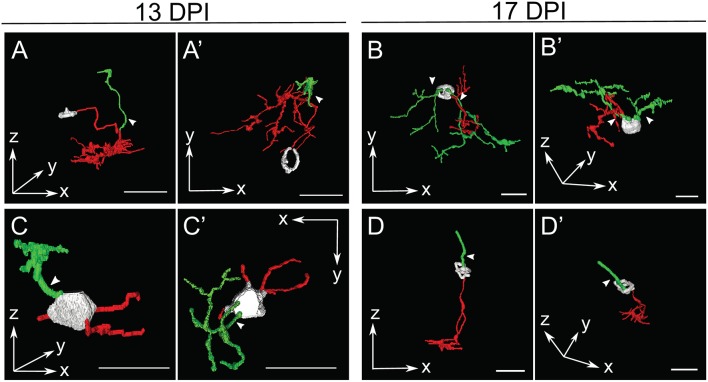
Selected retinal bipolar (BP) neurons that demonstrate the unusual range of morphologies observed at 13 and 17 days post-injury (DPI). Neurons were traced in Simple Neurite Tracer and then rendered in ImageJ's 3D viewer. **(A,A')** Neuron showing an apparently displaced soma and multiple neurites, shown from two different orientations (**A** = radial “side” view, **A'** = tangential “top-down” view). The cell body was only minimally traced due to limitations of SNT. **(B,B')** Neuron that has two distinct apparent dendritic trees connected to the same cell body. **(C,C',D,D')** Neurons with apparently simple morphologies sampled at 13 DPI and 17 DPI, respectively, each shown from two different orientations. In contrast to A and B, these BPs have a bipolar shape, but have smaller dendritic trees with fewer branches than most other neurons sampled at 13 and 17 DPI. The neuron in **(C,C')** appears to have more than one basal outgrowth (possibly multiple axons), all of them truncated, while the neuron in D has an axon with a normal appearance, but a single apical process rather than a dendritic tree. The cell body in **(D,D')** was only minimally traced due to limitations of SNT. Arrows designate investigator assignment of primary dendrites (green). Axons, red; incompletely traced cell bodies, grayscale. Scale bar(s) in all panels = 10 μm.

In several cases, the morphologies of newly regenerated BPs in 13 DPI and 17 DPI retinas were highly atypical (arrows in Figures 3, 5; Supplementary Table 2; Supplementary Figure 2). For example, one BP displayed a single major neurite that then branched into both apical and basal projections, sometimes with extensive branching (Figures 5A,A'), while others (three of 15 BPs at 13 DPI and two of 13 BPs at 17 DPI) displayed numerous neurites projecting from the cell body (Figures 5B,B',C,C'; Supplementary Table 2). Some of the BPs displayed neurites that were intertwined and seemingly lacked polarity with respect to the surrounding retinal tissue (Figures 5B,B'; Supplementary Table 2). Two of 15 BPs at 13 DPI and two of 13 BPs at 17 DPI showed highly truncated dendritic trees, visible only as short primary dendrites (Figures 5D,D'; Supplementary Table 2; Supplementary Figure 2C). Furthermore, some of the BPs displayed dendrites having secondary branches, even though the primary dendrite did not reach the OPL (one of 15 at 13 DPI and two of 13 at 17 DPI; Figure 5B; Supplementary Figure 2B). This type of feature has not been reported in regenerated retinas at 60 DPI (McGinn et al., 2018), in adult undamaged retina (Li et al., 2012; McGinn et al., 2018), or in developing zebrafish retina (Schroeter et al., 2006), and so may be distinct to newly regenerated BPs. Two of the 15 axons of newly regenerated BPs studied at 13 DPI displayed long, wandering axons (Figures 3, 4). Only seven of 15 BPs at 13 DPI displayed a single axon with one to three branching points (Supplementary Table 2) and could be considered stereotypical BP neurons (Li et al., 2012). A summary of BP numbers at each time point displaying characteristics not meeting criteria defining a “stereotypical appearance” of a BP (see Methods), is provided in Supplementary Table 2. These atypical morphologies, found far more frequently at 13 and 17 DPI than at 21 DPI, may represent immature stages of BP differentiation, steps in the process of axon and dendritic tree pathfinding, and/or abnormal regenerated neurons that may later be eliminated by cell death or clearance. It is also important to note that the relative numbers of morphologically abnormal-appearing BPs may be the consequence of uncontrollable variables such as transgene silencing, and/or by unavoidable potential biases in the selection of isolated, *nyx::mYFP*+ neurons for tracing. Because many of these morphologically abnormal neurons were difficult to unambiguously trace, and/or did not display the process branching characteristics required for the further morphometric analyses, they were not included in the following more detailed quantitative studies of dendritic trees and axonal branching patterns.

### Morphometric Analyses of Dendritic Trees of Newly Regenerated *nyx::mYFP* BPs

#### Dendritic Spread

**Figure 6:**
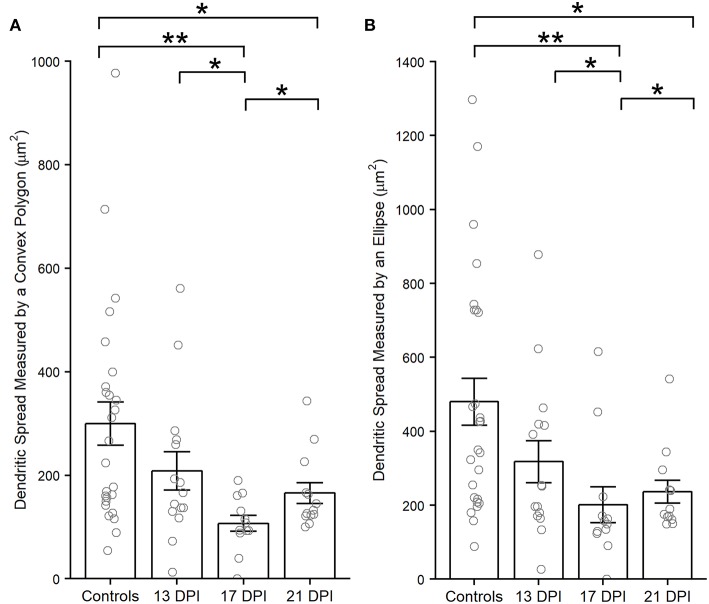
Dendritic spreads measured using the convex polygon method **(A)** and the ellipse method **(B)**, of regenerated *nyx::mYFP*+ bipolar (BP) neurons, are reduced at 17 and 21 days post-injury (DPI) compared to controls. Column graphs show the mean of each group, open circles represent each individual, measured dendritic tree, and the error bars show the SEM. *p < 0.05, **p < 0.01 using Wilcoxon–Mann–Whitney tests. Control measurements are those shown in McGinn et al. (2018).

Dendritic spreads of several (Supplementary Table 1) traced, regenerated nyx::YFP+ BPs were measured using an ellipse or polygonal method (see Methods). Interestingly, the dendritic spreads of regenerated BPs at 13 DPI were not different than those of control BPs (*p* = 0.216, measured by a convex polygon; *p* = 0.072, measured by an ellipse) (Figure 6). However, the dendritic spreads of BPs in the 17 DPI and 21 DPI retinas were smaller than those in control retinas (*p* = 0.002 and *p* = 0.038, complex polygon; *p* = 0.004 and *p* = 0.016, ellipse) (Wilcoxon–Mann–Whitney) (Figure 6).

#### Dendritic Tree Characteristics

**Figure 7:**
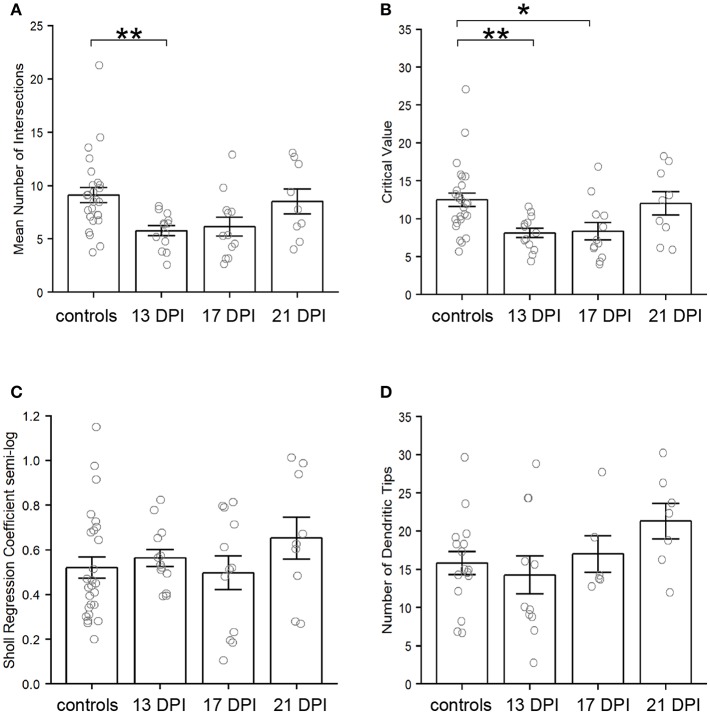
Characteristics of *nyx::mYFP*+ bipolar (BP) neuron dendritic trees. **(A–C)** Outputs of Sholl analysis. **(A)** Average number of intersections is reduced at 13 days post-injury (DPI) but not at 17 and 21 DPI in comparison with controls. B. Sholl critical values are reduced at 13 and 17 DPI but not at 21 DPI in comparison with controls. C. Sholl regression coefficients are not significantly different across samples. **(D)** Numbers of dendritic tips (presumed photoreceptor connections) are not significantly different across samples. Bar graphs show the mean of each group, open circles represent each individual dendritic tree, and the error bars show the SEM. *p < 0.05, **p < 0.01 using Wilcoxon–Mann–Whitney tests. Control measurements are those shown in McGinn et al. (2018).

Using the Sholl Analysis plugin in ImageJ, the extent of dendritic branching was calculated separately for several individual neurons (see Methods; Supplementary Table 1). A “mean of intersections” was returned by the plugin, a measure which is calculated by dividing the sum of the number of dendrite crossings of each concentric sphere by the number of concentric spheres (Ferreira et al., 2014). A higher value for mean of intersections indicates more extensive branching. This measure of dendritic branching was lower for BPs in 13 DPI retinas than for those in control retinas (*p* = 0.006) (Figure 7A). However, the extent of dendritic branching reached control levels for regenerated BPs sampled at 17 DPI and 21 DPI (*p* = 0.054 and *p* = 0.883) (Figure 7A). Therefore, while the sizes of the dendritic trees of 13 DPI BPs were similar to those of controls (Figure 6), these trees were less extensively branched, a finding that could be considered consistent with dendrite restructuring toward presynaptic partners of regenerated BPs. Results from Sholl analysis also revealed significant reductions in the Sholl critical value for 13 DPI and 17 DPI BPs as compared with controls (*p* = 0.0088 and *p* = 0.0237), but dendrites of 21 DPI BPs had Sholl critical values matching those of controls (*p* = 0.921) (Figure 7B). The Sholl critical value is the distance at which the maximum number of dendritic crossings is found, and so these results indicate that the BPs sampled at earlier regeneration times likely had fewer dendritic branches at the greater distances from the primary dendrite.

As a further measure of overall dendritic morphology, the Sholl regression coefficient was calculated, using the semi-log method; the regression coefficient can be used to determine if neurons fall into the same broad morphological category (Ristanovic et al., 2006). A one-way ANOVA across all sampling times revealed no significant differences (*p* = 0.444) (Figure 7C), indicating that the dendritic trees of newly regenerated BPs were not sufficiently distinct from those of control BPs as to constitute a separate morphological neuronal type.

### Analyses of Dendritic Tips and Cone Contacts of Newly Regenerated *nyx::mYFP* BPs

#### Dendritic Tips

Endpoints (dendritic tips, which project apically toward their presynaptic photoreceptor partners) of *nyx::mYFP* BPs were counted in regenerated retinas at 13, 17, and 21 DPI and compared to controls (McGinn et al., 2018) (Supplementary Tables 1, 3). The average total numbers of dendritic tips per BP showed no statistically significant differences across all conditions (*p* = 0.194, one-way ANOVA.) (Figure 7D). It is important to again note, however, that neurons with highly truncated dendritic trees (e.g., Figure 5D), were excluded from this analysis because they had no endpoints to count, and so these numbers of dendritic tips do not represent the entire BP population sampled.

#### Cone Contacts

**Figure 8:**
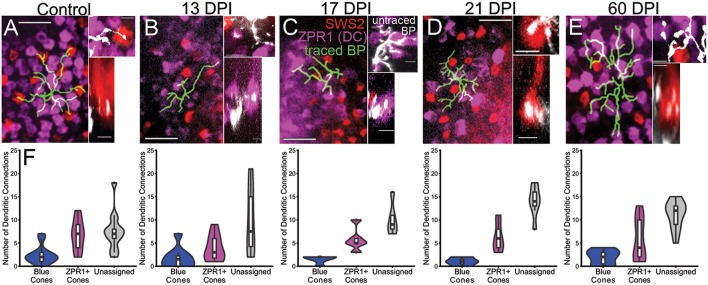
Reestablishment of *nyx::mYFP*+ bipolar (BP) neuron dendritic connections with cone photoreceptor terminals. **(A–E)** Dendritic fields showing traced *nyx::mYFP*+ BPs (green) overlayed onto partial projections of blue-sensitive (red color) cone terminals and ZPR1+ (magenta color) red- and green-sensitive double cone terminals, from control **(A)**, 13 days post-injury (DPI) **(B)**, 17 DPI **(C)**, 21 DPI **(D)**, and 60 DPI **(E)** retinas. Control and 60 DPI images also appear in McGinn et al. (2018). Higher magnification views on right of each panel show untraced BP dendrites (white) forming connections with blue-sensitive (red color) and/or ZPR1+ (magenta color) red- and green-sensitive double cone terminals (Top images are z-projections; bottom images are partial projections from image stacks, showing resliced radial views; control and 60 DPI images also appear in McGinn et al. (2018), but with different pseudocoloring). **(F)** Distributions of dendritic connections to identified and unassigned photoreceptor subtypes for *nyx::mYFP*+ BPs in control, 13 DPI, 17 DPI, 21 DPI, and 60 DPI retinas. Shapes of violin plots were obtained by using a kernel density estimator to generate a smoothened histogram, mirrored along the x-axis, and then rotated. The width of each plot is determined by the proportion of bipolar cells making a given number of connections to that photoreceptor subtype at that point. In the boxplots within, the horizontal line inside each box represents the median, the top and bottom of the box represent the 25th and 75th percentiles, the whiskers represent the 1.5 interquartile range, and the filled circles represent outliers. Control vs. 13 DPI *p* = 0.3844, control vs. 17 DPI *p* = 0.2758, control vs. 21 DPI *p* = 0.031, control vs. 60 DPI *p* = 0.4534 (Generalized linear model). Scale bars in **(A–E)** = 10 μm for left panels, and 2.5 μm for right panels.

Traced, *nyx::mYFP* BP dendritic tips were evaluated for the presence of presumptive synaptic connections with cone photoreceptors. To this end, we simultaneously visualized mCherry+ synaptic terminals of blue-sensitive cones, and ZPR1-antibody labeled synaptic terminals of double cones (a.k.a. red- and green-sensitive cones) (Figures 8A–E; Supplementary Table 3) in whole *nyx::YFP* transgenic retinas. For this analysis we again excluded BPs that did not reach the OPL with their apical dendrites. The patterns of connections, measured as the proportion of endpoints contacting blue cones vs. double cones vs. unassigned endpoints (which could represent UV cones, rods, or unconnected tips), were quantified (Figure 8F; Supplementary Table 3). In our previous study, ZPR1+ cone pedicles could readily be identified as belonging to green-sensitive or red-sensitive cones based upon their location with respect to surrounding cone terminals of the organized mosaic (McGinn et al., 2018). Unfortunately, we were unable to unambiguously make these specific assignments as the mosaic of cone terminals at 13, 17, and 21 DPI was difficult to appreciate, perhaps due to tissue fragility (Figures 8B–D). Instead, each regenerated BP dendritic endpoint was counted as connecting to a blue sensitive cone, a ZPR1+ cone, or unassigned. We also re-analyzed cone contacts observed in control retinas, and at 60 DPI (McGinn et al., 2018) according to these criteria (Figure 8F; Supplementary Table 3). We then applied a Generalized Linear Model (GLM) to determine whether BP-cone connectivity patterns were different in control vs. regenerated retinas (i.e., do regenerated *nyx::mYFP* BPs sampled at any timepoint connect more or less frequently to any specific cone subtype than in control retina?). Based on this analysis, regenerated BPs sampled at 13, 17 DPI, and 60 DPI each showed patterns of photoreceptor connections that were statistically indistinguishable from controls (*p* = 0.3844, 0.2758, and 0.4534, respectively; GLM) (Figure 8F). Interestingly, the BPs sampled at 21 DPI were significantly different than controls (*p* = 0.031), apparently favoring ZPR1+ double cone contacts over blue cone contacts (Figure 8F), suggesting some plasticity of synaptic connections during regeneration.

Although the above analysis suggests that relatively normal connectivity may be restored at 13 and 17 DPI, it is again important to note that not all bipolar cells reconnected with visualized photoreceptors at these sampling times. Neurons with 1 or 0 projections reaching the OPL were excluded from the connectivity analysis due to the ambiguity of such connections.

### Axon Morphologies of Newly Regenerated BPs

We previously analyzed BP axons in regenerated retinas (at 60 DPI) through identification of stratification patterns and Sholl analysis of complexity of axon terminals (McGinn et al., 2018). In several cases in the present study, the IPL was difficult to clearly identify at 13, 17, and 21 DPI, and we were therefore unable to unambiguously subdivide the vague landmarks defining the IPL into the six typical substrata (Connaughton and Nelson, 2000), precluding analysis of axonal stratification. As discussed above, some BP axons observed in 13, 17, and 21 DPI regenerated retinas traversed long distances, branched into inappropriate retinal layers, or appeared to abruptly terminate (Figures 3–5, Supplementary Table 2; Supplementary Figure 2). Such axons were difficult and at times impossible to completely trace because axons would continue to traverse beyond the imaged region. Some of the more unusual-appearing BP axons from regenerating retina (which were traceable) were examined in further detail, and two are provided as 3-D projections in order to appreciate the context of each BP axon and surrounding regenerating retinal tissue (Supplementary Figures 2B,D), particularly in comparison with a BP axon residing within undamaged retina (Supplementary Figure 2A). One of these BPs had a cell body residing at the boundary of the GCL rather than within the INL (Supplementary Figure 2B), an abnormal position for this cell type that we previously documented for the rare BP sampled at 60 DPI (McGinn et al., 2018).

**Figure 9:**
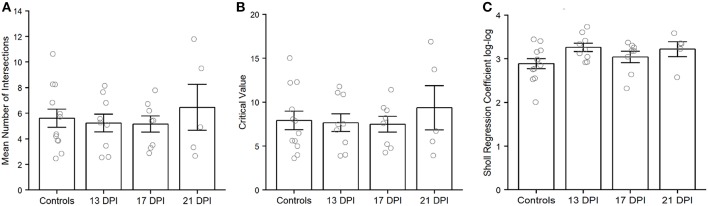
Characteristics of *nyx::mYFP*+ bipolar (BP) neuron axonal arbors, using outputs of Sholl analysis. Average number of intersections **(A)**, Sholl critical values **(B)**, and Sholl regression coefficients **(C)** are not significantly different across samples. Column graphs show the mean of each group, open circles represent each individual dendritic tree, and the error bars show the SEM. Wilcoxon-Mann-Whitney tests did not reveal any significant differences for any of these measures. Controls are those shown in McGinn et al. (2018). DPI, days post-injury.

Because we were unable to completely trace many of the axons of newly regenerated BPs, we were not able to unambiguously measure axonal length as a potential metric of our qualitative observations. As an alternative, but distinctive analysis of axon characteristics, we performed Sholl analysis of axonal branching patterns for some of the BPs sampled from 13, 17, and 21 DPI retinas (Supplementary Table 1). The average number of intersections returned from Sholl analysis of axon branching indicated no significant differences among samples (*p* = 0.8) (Figure 9A). Similarly, the Sholl critical value reached control levels for regenerating BPs sampled at all regenerating time points (*p* = 0.981) (Figure 9B).The Sholl regression coefficient also showed no significant differences among samples (*p* = 0.12) (Figure 9C). These measureable axons therefore displayed axon branching characteristics similar to those of mature BPs.

## Discussion

### Emergence of Regenerated BP Neurons

In this study, we report the neuronal morphologies and cone photoreceptor connectivities of regenerated BPs in adult zebrafish retinas from 13 to 21 days after a ouabain-induced lesion destroying only inner retinal neurons. After such a lesion (Nagashima et al., 2013; Sherpa et al., 2014; McGinn et al., 2018; Mitchell et al., 2018, 2019), regenerated BPs must extend axons and dendrites that locate and connect to their input partners (photoreceptors), which survived the lesion, as well as their output partners (amacrine and ganglion cells), which were destroyed and also regenerated following the lesion. We find that new PKCα+ and *nyx::YFP*+ BPs initially emerge by 13 DPI in reduced numbers compared to undamaged retinas, and that numbers of the PKCα+ BPs at 21 DPI show no evidence of a difference with numbers of these neurons in control retinas. This pattern of emergence of BPs is consistent with protracted and asynchronous regeneration of BPs, and/or with asynchronous expression of BP markers. Our birthdating studies support the former interpretation, as they suggest steady production of both PKCα+ BPs and their postsynaptic partners, HuC/D+ amacrine and ganglion cells, over 6–13 DPI. Interestingly, this sequence of neuronal production during regeneration does not appear to recapitulate the sequence documented for embryonic retinal neurogenesis in the zebrafish, in which cells of the GCL are generated prior to those of the INL (Hu and Easter, 1999). Rather, during retinal regeneration, PKCα+ BPs and their post-synaptic partners are generated concurrently, and this regeneration occurs in a protracted manner, as has been shown for HuC/D+ amacrine and ganglion cells for even longer timeframes (Sherpa et al., 2014).

### General Attributes of Newly Regenerated BP Neurons

The morphologies of *nyx::mYFP*+ BPs sampled at 13, 17, and 21 DPI were in some cases highly unusual, with abnormal positions and sizes of apically and basally-projecting neurites, and/or laterally projecting axons, and/or no cone contacts. This is in contrast to BPs analyzed in our previous study of *nyx::mYFP*+ BPs, which were sampled at 60 DPI, in which the majority of sampled BPs showed morphologies indistinguishable from controls (McGinn et al., 2018). In the present study, only a subset of analyzed BPs displayed a rather stereotypical appearance, closely resembling BPs sampled from undamaged retinas. One reason for such morphological differences in BPs compared at these different timepoints could be that relatively earlier regenerated BPs may be in the process of establishing their mature morphologies, with further growth and/or pruning or retraction taking place over 17–21 DPI. During mouse retinal development, apical and basal processes of differentiating BPs must retract as they elaborate their dendritic trees and axonal arbors, respectively (Morgan et al., 2006). Our staining and imaging procedures did not reveal, and perhaps may not be able to reveal, the presence of initial apical or basal processes that span the retina, as was found in mouse, but our findings do suggest regenerated zebrafish BP neurites may undergo pathfinding which extends beyond their potential targets, followed by refinement. Alternatively, mature BP morphologies may be manifest concomitantly with expression of the markers used to identify them in this study, and those BPs showing abnormal morphologies may be eliminated by cell death or clearance. Another possible explanation is that protracted regeneration of BPs and other inner retinal neurons may create a tissue environment that allows for more efficient neurite pathfinding over time (for example, due to increased or better organized spatial and directional cues), resulting in the later-regenerated BPs appearing more morphologically normal. We acknowledge that the PKCα+ BPs analyzed for birthdating represent a different population than the *nyx::mYFP*+ BPs we analyzed morphometrically (McGinn et al., 2018), and that both populations are heterogeneous (Schroeter et al., 2006; McGinn et al., 2018). Therefore, the protracted regeneration process of PKCα+ BPs may not be reflected by a similar time course for the *nyx::mYFP*+ BPs, and the apparent maturation dynamics and aberrant morphologies of the *nyx::mYFP*+ BPs may not be manifested by newly regenerated PKCα+ BPs. Finally, the heterogeneity of morphologies of newly regenerated *nyx::mYFP*+ BPs may represent distinct maturation strategies for different subpopulations of these BPs.

### Dendritic Attributes of Newly Regenerated BP Neurons

When measureable, BP dendritic tree sizes appeared to fluctuate over the 13–21 DPI timeframe. It is possible that this timeframe (and perhaps beyond) represents a time of modification of dendritic processes. Interestingly, dendritic tree attributes of the newly regenerated BPs also showed some statistically significant differences in comparison with controls, but dendrite attributes from BPs sampled at 21 DPI showed the most normal morphologies. For example, Sholl critical values of the 13 DPI and 17 DPI, but not of the 21 DPI, BP dendritic trees, were different from the controls. This suggests that dendritic branches are present at 13 DPI but may continue to be modified until 21 DPI, when they approach a more stable state. Similarly, the mean number of intersections was reduced for BP dendrites at 13 DPI, but not for those sampled at 17 DPI or 21 DPI. These results suggest that regenerating *nyx::mYFP* BPs sampled at 13 DPI have dendritic trees with fewer branches, shorter branches, or a combination of both, as compared with other sampling conditions. In general, the outputs of Sholl analysis indicate that the dendritic trees of sampled, regenerated BPs may undergo morphological changes consistent with maturation over the 13–21 DPI sampling time. It is interesting that this possible maturation process appears distinct from that of BP dendritic trees in developing retina. For example in mouse, BP dendritic field sizes are initially overextended and overlapping with each other, and then retract due to homotypic interactions with neighboring BPs (Lee et al., 2011). In larval zebrafish, developing *nyx::mYFP*+ BPs have also been observed to overextend their dendritic trees, but into the ONL and INL, rather than within the plane of the OPL (Schroeter et al., 2006).

Not all BPs showed visible connections to photoreceptors at 13 and 17 DPI. However, those that did, showed cone connectivities similar to those of control BPs. In addition, the numbers of presumed cone contacts (dendritic tips) were statistically similar across all timepoints. Together with the shifting dendritic field sizes and complexities discussed above, we hypothesize that regenerating BPs may make their connections to photoreceptors before their dendritic trees are mature, an event that could be likely in contexts where pre-synaptic photoreceptors are present during BP regeneration. In comparison, following cell-selective ablation of distinct subpopulations of BPs in larval retina, regenerated BPs display overall normal morphologies, but have larger dendritic trees and some errors in cone contacts (D'Orazi et al., 2016). It is possible that these regenerating larval BPs were analyzed prior to achieving a more mature state, as they were sampled only 13 days after ablation, and may represent a parallel sampling to the possibly immature BPs we describe here at 13 and 17 days after ouabain injury in adult fish. Similar phenomena have been described during embryonic retinal development in both mouse (Morgan et al., 2006) and zebrafish (Schroeter et al., 2006), in which the dendritic trees of differentiating BPs also appear to grow, elaborate beyond their intended targets, and then retract to their stable arbor morphologies.

An alternative interpretation for the observed differences in BP dendritic tree morphologies at 13 vs. 17 vs. 21 DPI, is that some of the regenerated BPs may undergo apoptosis or are cleared from the retina (or are less likely to be sampled), while new BPs continue to be generated and assume normal morphologies and connectivities. BP cell death alone as the explanation for the apparent loss of abnormal BPs between 17 and 21 DPI is somewhat unlikely for several reasons. Numbers of TUNEL+ cells in retinas over this timeframe are minimal compared to the initial (1–3 DPI) period of apoptosis due to ouabain damage (Fimbel et al., 2007). Furthermore, quantification of BPs suggests their accumulation rather than loss (Figure 1). Finally, Hitchcock and Cirenza (1994) found that amacrine cells were able to remodel and reintegrate into regenerated portions of the retina following surgical excision of a portion of goldfish retina. It is possible that regenerated BPs possess the same ability as regenerated amacrine cells to remodel or change their morphology accordingly.

### Axonal Attributes of Newly Regenerated BP Neurons

Among the more noteworthy abnormalities observed in the newly regenerated BPs were the unusual trajectories of some axons. In many cases axons traversed such long distances and/or entered an inappropriate layer, making complete documentation of their trajectories difficult or impossible. At the other extreme, some of the regenerated BPs displayed what appeared to be more than one basally-projecting neurite, or no basally-projecting neurite at all. These abnormalities were more frequently observed in the BPs sampled at 13 and 17 DPI in comparison with those at 21 DPI (Supplementary Table 2), and so may again represent immature states of the regenerated BPs prior to pruning/retraction or growth. Also noteworthy is that the newly regenerated BPs that could be sampled for Sholl analysis demonstrated axonal branching characteristics not significantly different from those of control BPs. One interpretation of these more normal BP axons is that they may represent those of the earliest-generated and therefore potentially more mature BPs. Such an interpretation is consistent with the findings for BP axon development in zebrafish larvae, in which active extension and retraction of axon filopodia takes place prior to the establishment of axon terminals in their appropriate sublaminae (Schroeter et al., 2006). Alternatively, the more normal axons may reside within an environment in which at least some of their postsynaptic partners have been more extensively regenerated, while the abnormal BP axons reside in regions with (or are regenerated at timepoints that display) a paucity of postsynaptic targets and plexiform structure, and lack the pathfinding cues that would guide them to the correct retinal location and synaptic partners. In support of this alternative, the absence of retinal ganglion cells and disruption of amacrine cell stratification during zebrafish larval development results in mis-targeting of BP axons (Kay et al., 2001).

## Conclusions

Following a lesion that destroys BPs and their postsynaptic partners, but spares their presynaptic partners, newly regenerated BPs are generated over a protracted timeframe and reappear from 13 to 21 DPI. Newly regenerated BPs display morphological characteristics consistent either with dynamic modifications of their dendritic and axonal arbors, or with heterogeneity in regenerated BPs dependent upon the time of regeneration, or some combination of these interpretations. In any case, generation and morphological differentiation of regenerated BPs is not a simple recapitulation of these processes during embryonic development, but distinctive to regeneration. Further, our data indicate that regeneration remains a dynamic process, as opposed to a single burst of genesis to replace lost neurons. Since photoreceptors are spared from this lesion, the presence and appropriate locations of photoreceptor synaptic terminals in regenerating retinas may provide long- or short-range cues to attract and pair with regenerated BP dendrites. The photoreceptors that survive this lesion have the capacity to support the a-wave of the electroretinogram (ERG) (McGinn et al., 2018), indicating that they remain physiologically active, and this activity may also provide targeting information for BP dendrites. In contrast, the absence or concomitant emergence of post-synaptic ganglion and amacrine cell dendrites leaves BP axons with potentially inappropriate (or absent) guidance information. These results are consistent with our previous study documenting faster recovery of visual function following inner retinal lesion compared to more extensive damage that includes photoreceptors (Sherpa et al., 2014).

Despite the presence of many morphologically abnormal regenerated BPs, the regenerated BPs that display dendritic trees and axons amenable to tracing and further analysis show characteristics that are quite similar to those of mature, undamaged BPs. Collectively our results suggest that the intrinsic mechanisms underlying the reestablishment of retinal circuitry during regeneration are robust, even in the context of a tissue-disrupting lesion. Further, the 13–21 DPI period following an inner retina-selective lesion appears to be an active time of both BP neurogenesis and the reestablishment of retinal circuitry. Among the next steps in the study of regeneration of retinal neurons and their connections will be to identify the cellular signaling and other processes that support functional rewiring (Angueyra and Kindt, 2018), as well as to determine the extent to which regenerated neurons are required in numbers and morphological “normalcy” to restore measureable physiological function. An evaluation of cell-specific transcriptomes at this time post-lesion may reveal signals that are specific for this rewiring (Sun et al., 2018a,b). In addition, microglia remain active over this period following retinal damage and regeneration (Mitchell et al., 2018), and have known roles in synaptic remodeling in other biological contexts (Schafer et al., 2012), making them excellent candidates as participants in the reestablishment of circuitry in the regenerated retina.

## Ethics Statement

This study was carried out in accordance with the recommendations of the Guide for the Care and Use of Laboratory Animals. The protocol was approved by the Animal Care and Use Committee of the University of Idaho.

## Author Contributions

TM and DS conceived of the study. TM, DM, and DS planned the experiments. TM, CG, and DM carried out the experiments and analyzed data. DL and NP contributed to data analysis. TM, DM and DS wrote the manuscript with approval of all authors.

### Conflict of Interest Statement

The authors declare that the research was conducted in the absence of any commercial or financial relationships that could be construed as a potential conflict of interest.
