# Supplementary material for: Rewiring the Regenerated Zebrafish Retina: Reemergence of Bipolar Neurons and Cone-Bipolar Circuitry Following an Inner Retinal Lesion
Source: Front Cell Dev Biol. 2019 Jun 6;7:95. doi: 10.3389/fcell.2019.00095 (PMC6562337; doi:10.3389/fcell.2019.00095)
Supplement: Supplementary file 1 [file Table_1.docx]

**Supplemental Table 1.** **Numbers of neurons used for visualization and/or morphometric analyses from each retina.**

| **Retina ID** | **Condition** | **Traced** | **Rendered** | **Dendritic Spread** | **Sholl (Dendrites)** | **Dendritic Tips** | **Cone Contacts** | **Sholl (Axons)** |
| --- | --- | --- | --- | --- | --- | --- | --- | --- |
| 1 | Control^1^ | 1 |  | 1 | 1 |  |  |  |
| 2 |  | 4 | 1 | 4 | 4 |  |  | 1 |
| 3 |  | 4 | 4 | 3 | 3 | 3 | 3 | 3 |
| 4 |  | 4 |  | 4 | 2 |  |  |  |
| 5 |  | 1 | 1 | 1 | 1 | 1 | 1 | 1 |
| 6 |  | 2 | 1 | 2 | 2 | 1 | 1 | 1 |
| 7 |  | 3 | 2 | 3 | 3 | 3 | 3 | 2 |
| 8 |  | 1 | 1 | 1 | 1 | 1 | 1 | 1 |
| 9 |  | 4 | 2 | 4 | 4 | 4 | 4 | 3 |
| 10 |  | 3 |  | 3 | 3 | 3 |  |  |
| 11 |  | 1 | 1 |  |  |  |  |  |
| 17 | 13 DPI^2^ | 2 | 2 | 2 | 2 | 2 | 1 |  |
| 18 |  | 2 | 2 | 2 | 2 | 2 | 2 | 2 |
| 19 |  | 2 | 1 | 1 |  |  |  |  |
| 20 |  | 0 |  |  |  |  |  |  |
| 21 |  | 6 | 5 | 6 | 6 | 4 | 2 | 6 |
| 22 |  | 3 | 1 | 3 | 3 | 3 | 3 | 1 |
| 24 | 17 DPI | 3 | 3 | 2 | 2 |  |  | 2 |
| 25 |  | 3 | 2 | 3 | 3 |  |  | 1 |
| 26 |  | 2 | 2 | 1 | 1 |  |  | 1 |
| 27 |  | 2 | 2 | 2 | 2 | 2 | 1 | 1 |
| 28 |  | 4 | 4 | 4 | 4 | 4 | 4 | 3 |
| 29 | 21 DPI | 2 | 2 |  |  |  |  |  |
| 31 |  | 4 |  | 4 | 1 |  |  | 1 |
| 32 |  | 3 | 3 | 3 | 2 | 3 | 3 |  |
| 34 |  | 3 | 3 | 3 | 3 | 2 | 2 | 3 |
| 35 |  | 4 | 4 | 3 | 3 | 2 | 2 | 1 |

^1^ Control neurons also reported in [4].

^2^ DPI, days post-injury.
