# Supplementary material for: Rewiring the Regenerated Zebrafish Retina: Reemergence of Bipolar Neurons and Cone-Bipolar Circuitry Following an Inner Retinal Lesion
Source: Front Cell Dev Biol. 2019 Jun 6;7:95. doi: 10.3389/fcell.2019.00095 (PMC6562337; doi:10.3389/fcell.2019.00095)
Supplement: Supplementary file 2 [file Table_2.docx]

**Supplemental Table 2. Numbers of bipolar (BP) neurons with abnormal morphologies vs. total analyzed for specific features.**

| **Condition** | **# BPs analyzed for abnormalities** | **BPs with endpoints not reaching OPL** | **BPs with meandering axons** | **BPs with >2 neurites and/or unusual polarity** | **BPs with other abnormal morphologies^1^** |
| --- | --- | --- | --- | --- | --- |
| **Control** | 25 | 0 (0%) | 0 (0%) | 0 (0%) | 0 (0%) |
| **13 DPI^2^** | 15 | 1 (6.7%) | 2 (15.4%) | 3 (20%) | 3 (20%) |
| **17 DPI** | 13 | 3 (23.1%) | 0 (0%) | 2 (15.4%) | 6 (46.2%) |
| **21 DPI** | 13 | 0 (0%) | 0 (0%) | 1 (7.7%) | 0 (0%) |

^1^ Other abnormal morphologies: highly truncated dendritic trees, dendrites with secondary branches, even though primary dendrite did not reach outer plexiform layer.

^2^ DPI, days post-injury.
