# Supplementary material for: Rewiring the Regenerated Zebrafish Retina: Reemergence of Bipolar Neurons and Cone-Bipolar Circuitry Following an Inner Retinal Lesion
Source: Front Cell Dev Biol. 2019 Jun 6;7:95. doi: 10.3389/fcell.2019.00095 (PMC6562337; doi:10.3389/fcell.2019.00095)
Supplement: Supplementary file 3 [file Table_3.docx]

**Supplemental Table 3. Numbers of dendritic endpoints and specific cone contacts per bipolar (BP) neuron.**

| **Condition** | **BP ID#** | **Total Endpoints** | **Blue Cone Contacts** | **ZPR1+^1^ Cone Contacts** | **Unassigned Endpoints** |
| --- | --- | --- | --- | --- | --- |
| **Control** | 6 | 24 | 2 | 11 | 11 |
|  | 7 | 17 | 7 | 3 | 7 |
|  | 8 | 30 | 3 | 9 | 18 |
|  | 13 | 18 | 6 | 4 | 8 |
|  | 15 | 8 | 2 | 2 | 4 |
|  | 16 | 19 | 1 | 10 | 8 |
|  | 17 | 15 | 2 | 7 | 6 |
|  | 18 | 7 | 1 | 3 | 3 |
|  | 19 | 20 | 1 | 12 | 7 |
|  | 21 | 15 | 0 | 7 | 8 |
|  | 22 | 15 | 2 | 7 | 6 |
|  | 23 | 18 | 2 | 6 | 10 |
|  | 20 | 12 | 3 | 7 | 2 |
|  | 26 | 14 | N.Q.^2^ | | |
|  | 24 | 14 | N.Q. | | |
|  | 25 | 7 | N.Q. | | |
| **13 DPI^3^** | 56 | 10 | 2 | 6 | 2 |
|  | 57 | 9 | N.Q. | | |
|  | 58 | 24 | 1 | 9 | 14 |
|  | 59 | 10 | 2 | 3 | 5 |
|  | 61 | 29 | 7 | 1 | 21 |
|  | 62 | 9 | N.Q. | | |
|  | 63 | 16 | N.Q. | | |
|  | 66 | 16 | 3 | 3 | 10 |
|  | 67 | 3 | 0 | 1 | 2 |
|  | 68 | 24 | 0 | 6 | 18 |
|  | 69 | 7 | 0 | 2 | 5 |
| **17 DPI** | 77 | 14 | N.Q. | | |
|  | 78 | 13 | 2 | 3 | 8 |
|  | 79 | 14 | 0 | 5 | 9 |
|  | 80 | 14 | 2 | 5 | 7 |
|  | 81 | 28 | 2 | 10 | 16 |
|  | 82 | 19 | 2 | 6 | 11 |
| **21 DPI** | 87 | 26 | 0 | 11 | 15 |
|  | 88 | 16 | 0 | 4 | 12 |
|  | 89 | 30 | 2 | 10 | 18 |
|  | 90 | 24 | 1 | 6 | 17 |
|  | 92 | 19 | 1 | 4 | 14 |
|  | 94 | 22 | 2 | 6 | 14 |
|  | 95 | 12 | 1 | 3 | 8 |
| **60 DPI** | 27 | 11 | N.Q. | | |
|  | 28 | 17 | N.Q. | | |
|  | 29 | 17 | N.Q. | | |
|  | 30 | 7 | N.Q. | | |
|  | 31 | 10 | N.Q. | | |
|  | 32 | 8 | N.Q. | | |
|  | 39 | 7 | N.Q. | | |
|  | 40 | 19 | N.Q. | | |
|  | 41 | 21 | 4 | 2 | 15 |
|  | 42 | 15 | 3 | 2 | 10 |
|  | 43 | 18 | 2 | 4 | 12 |
|  | 44 | 26 | 0 | 13 | 13 |
|  | 45 | 20 | 0 | 7 | 13 |
|  | 46 | 19 | 4 | 10 | 5 |
|  | 47 | 25 | 2 | 11 | 12 |
|  | 48 | 25 | 1 | 10 | 14 |
|  | 50 | 16 | 3 | 1 | 12 |
|  | 51 | 20 | N.Q. | | |
|  | 52 | 28 | N.Q. | | |
|  | 49 | 20 | N.Q. | | |
|  | 53 | 13 | 3 | 2 | 8 |
|  | 54 | 9 | 0 | 1 | 8 |

^1^ ZPR1 labels green- and red-sensitive double cones.

^2^ N.Q., not quantifiable. Dendritic endpoints were too dim to unambiguously assign cone contacts.

^3^ DPI, days post-injury.
