# Supplementary figures and images for: Rewiring the Regenerated Zebrafish Retina: Reemergence of Bipolar Neurons and Cone-Bipolar Circuitry Following an Inner Retinal Lesion
Source: Front Cell Dev Biol. 2019 Jun 6;7:95. doi: 10.3389/fcell.2019.00095 (PMC6562337; doi:10.3389/fcell.2019.00095)

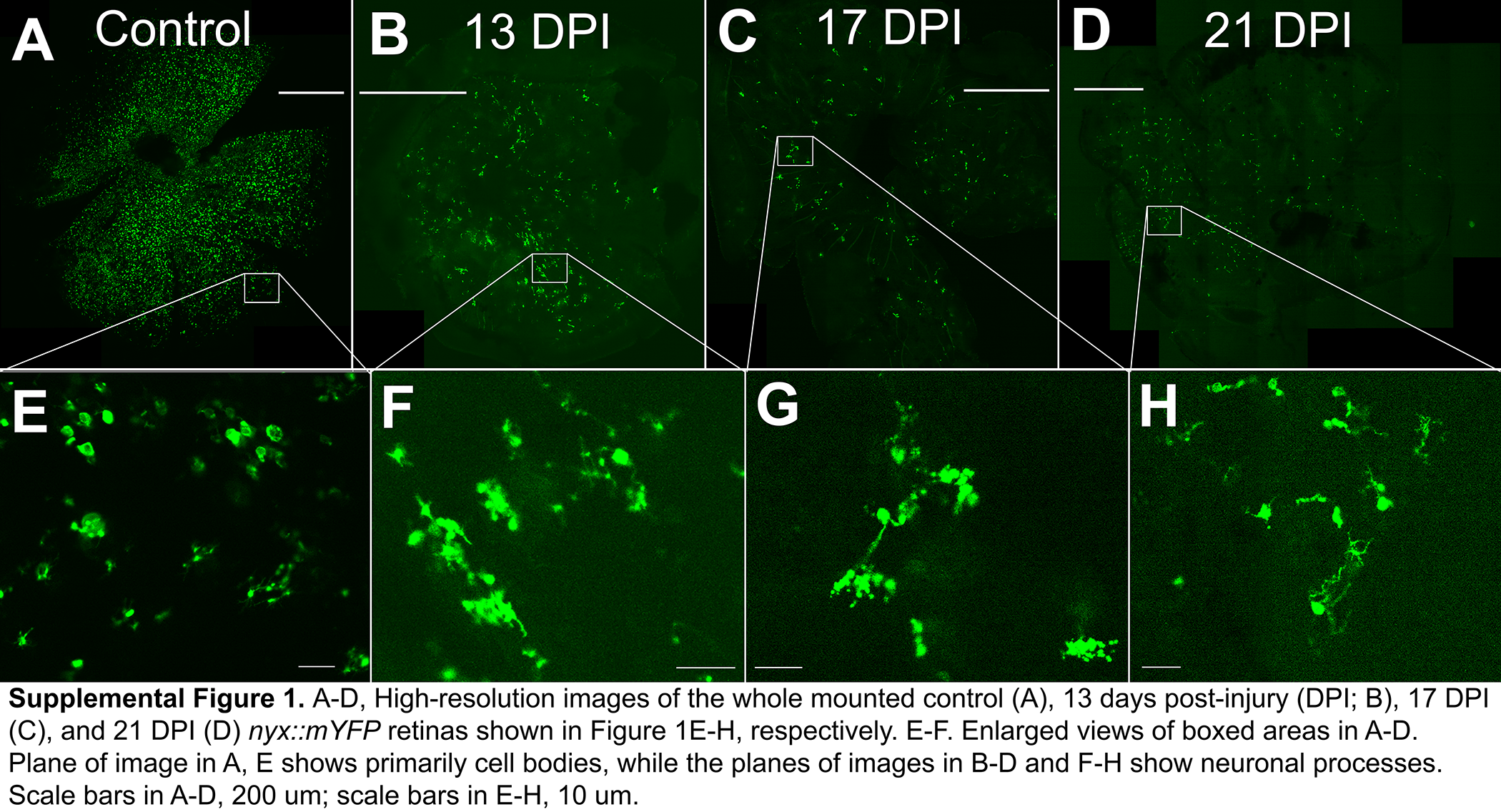

Supplement: Supplementary file 9 [file Image_1.TIF]

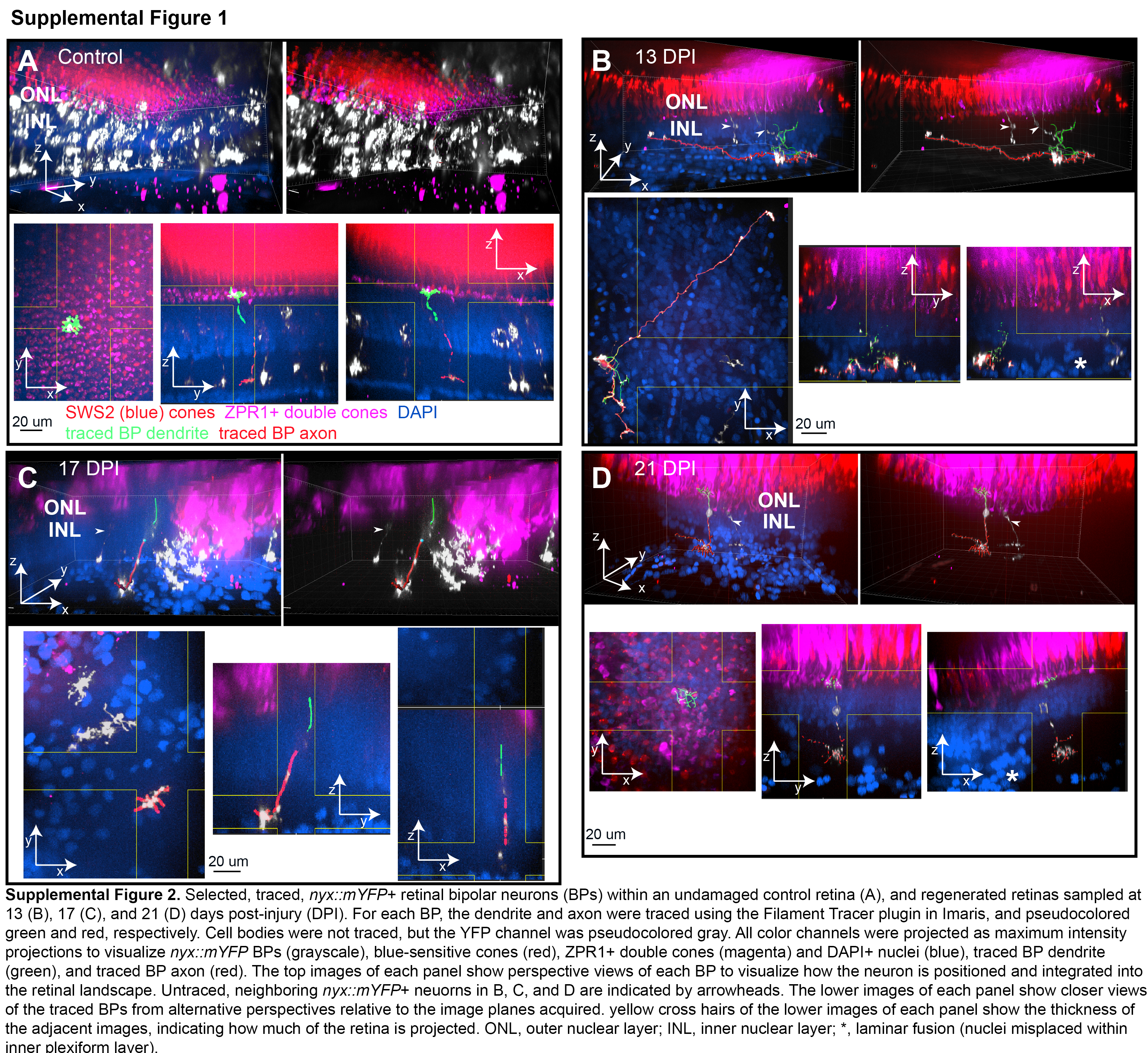

Supplement: Supplementary file 10 [file Image_2.TIF]
